# Untargeted MS-Based Metabolomic Analysis of Termite Gut-Associated Streptomycetes with Antifungal Activity against *Pyrrhoderma noxium*

**DOI:** 10.3390/antibiotics12091373

**Published:** 2023-08-28

**Authors:** Cherrihan Adra, Trong D. Tran, Keith Foster, Russell Tomlin, D. İpek Kurtböke

**Affiliations:** 1 School of Science, Technology and Engineering, University of the Sunshine Coast, Maroochydore BC, QLD 4558, Australia; cherrihan.adra@research.usc.edu.au (C.A.); ttran1@usc.edu.au (T.D.T.); 2 Centre for Bioinnovation, University of the Sunshine Coast, Maroochydore BC, QLD 4558, Australia; 3 Brisbane City Council, Program, Planning and Integration, Brisbane Square, Level 10, 266 George Street, Brisbane, QLD 4000, Australia; kfosteradsl@bigpond.com (K.F.); russell.tomlin@brisbane.qld.gov.au (R.T.)

**Keywords:** *Streptomyces*, streptomycetes, *Pyrrhoderma noxium*, termites, termite gut symbiosis, antifungal compounds, metabolomics, molecular networking, biological control, mass spectrometry

## Abstract

*Pyrrhoderma noxium* is a plant fungal pathogen that induces the disease of brown root rot in a large variety of tree species. It is currently infecting many of the amenity trees within Brisbane City of Queensland, Australia. Steering away from harmful chemical fungicides, biological control agents offer environmentally friendly alternatives. Streptomycetes are known for their production of novel bioactive secondary metabolites with biocontrol potential, particularly, streptomycete symbionts isolated from unique ecological niches. In this study, 37 termite gut-associated actinomycete isolates were identified using molecular methods and screened against *P. noxium*. A majority of the isolates belonged to the genus *Streptomyces*, and 15 isolates exhibited strong antifungal activity with up to 98.5% mycelial inhibition of the fungal pathogen. MS/MS molecular networking analysis of the isolates’ fermentation extracts revealed several chemical classes with polyketides being among the most abundant. Most of the metabolites, however, did not have matches to the GNPS database, indicating potential novel antifungal compounds in the active extracts obtained from the isolates. Pathway enrichment and overrepresentation analyses revealed pathways relating to polyketide antibiotic production, among other antibiotic pathways, further confirming the biosynthetic potential of the termite gut-associated streptomycetes with biocontrol potential against *P. noxium*.

## 1. Introduction

Invasive fungal pathogens pose an increasing threat to agriculture and food industries worldwide [1,2]. *Pyrrhoderma noxium* (*P. noxium*), formally known as *Phellinus noxius* [3], is a phytopathogenic fungus belonging to the family *Hymenochaetaceae* and the division Basidiomycota. It induces the disease of brown root rot, although it is classed as a white-rot fungus due to its ability to degrade lignin along with cellulose and hemicellulose [3]. This pathogen has caused much destruction throughout tropical and subtropical regions and is widespread throughout Taiwan [4,5,6], Japan [7,8], China [9,10], and Australia [11,12,13,14]. It is able to infect over 250 tree species, including herbaceous, broad-leaved, fruit, ornamental, and coniferous woody trees [15,16] within forests, landscapes, and orchards [17]. Infection begins within the roots and eventually extends to the basal stem [17]. Early stages of the disease are largely asymptomatic; therefore, diagnosis and treatment are usually hindered, and the tree health is too far declined for intervention [18]. Symptoms can include chlorosis, dieback, defoliation, and decay of stem and roots [19,20].

In response to the pressing need for sustainable and eco-friendly solutions to combat invasive fungal pathogens and reduce reliance on chemical fungicides, biological control methods have gained significant interest [21]. These methods involve the use of natural antagonists, such as functional microorganisms, to minimise the harmful effects of chemical agents used in conventional agriculture [22,23]. Among the diverse sources of natural antagonists, actinomycetes have emerged as prominent candidates as they are one of the largest producers of bioactive secondary metabolites and new antibiotics, with a recent focal shift on rare actinomycetes as an increasing target source for new secondary metabolites [24,25]. In fact, rare members of the genus *Streptomyces* persist to be the largest producer of new innovative metabolites [26]. Between 2015 and 2020, a significantly large number of novel *Streptomyces* spp. were isolated from various niches, such as extreme environments, sediments, marine environments, and symbionts [22,24]. Streptomycetes as microbial symbionts are distributed in a wide range of environments, including insect guts, cuticles, and nests [27]. Within this mutualistic relationship, streptomycetes assist insects in the breaking down of food, provide a synthesis of essential metabolites, alter gene descriptions, and combat pathogens and competitors [28]. One such source of these specialised streptomycetes symbionts is the guts of termites. Termites belong to the order Isoptera and have three main components of their alimentary tract: the foregut (stomodeum), the midgut (mesenteron), and the hindgut (proctodeum) [29]. They predominately feed on litter tissue and wood, or in other words, difficult-to-digest lignocelluloses; therefore, they depend on beneficial symbioses with a diversity of microflora for digestion and acquisition of supplementary nutrition [28]. Previous studies have revealed that actinomycetes are among the most dominant bacteria identified within the termite’s symbiotic lifestyle [29]. It is therefore possible that these unexplored insect guts may house rare species of actinomycetes with the ability to produce novel NPs [28,30]. To unravel the intricacies of these NPs in understudied streptomycetes, a metabolomic approach has proved to be a powerful tool [31]. Moreover, molecular networking [32] allows for the dynamic organisation of experimental spectra into families/classes based on spectral similarity with established MS/MS databases. It also provides visualisation and annotation of non-targeted mass spectrometry data [33,34].

In this study, 37 termite gut-associated actinomycetes selected from the University of the Sunshine Coast’s Microbial Library [35,36,37] were screened for antifungal activity against phytopathogen *P. noxium*. Their metabolic profiles were investigated using quadrupole time-of-flight (QTOF) mass spectrometry (MS) as well as molecular networking techniques for the purposes of screening and predicting bioactive molecules from the termite gut-associated actinomycetes active extracts and prioritising the best candidate(s) as potential biological control agents against phytopathogen *P. noxium*.

## 2. Results and Discussion

### 2.1. Phylogeny of Termite Gut-Associated Actinomycetes Based on the 16S rRNA Oligonucleotide Analysis

From the 37-termite gut-associated actinomycetes obtained from the UniSC’s Microbial Library, 32 of them (86%) had almost complete 16S rRNA gene sequences aligned with corresponding sequences of representative type strains of the genus *Streptomyces* (Tables S1 and S2). Five of the isolates (13%) aligned with the genus *Micromonospora* and one strain aligned with the genus *Saccharopolyspora* (Tables S1 and S2). Phylogenetic analysis demonstrated putative sequence identity percentage between 97 and 100% (Figure S1). The dominance of *Streptomyces* isolates within the termite gut aligned with previous studies and demonstrated the crucial role these bacteria play in termite symbiosis [38,39]. One study analysed the phylogeny of 118 actinomycete isolates collected from three types of termite nests (mound, carton, and subterranean nests) and determined *Streptomyces* spp. to be the most dominant genus accounting for approximately 60% of the isolates [40]. Another study completed a 16S analysis of 16 actinomycetes isolates from termite guts and found 9 of them aligned with the genus *Streptomyces* [41].

### 2.2. Screening for Antifungal Activity from Termite Gut-Associated Actinomycete Isolates against P. noxium

**Figure 1:**
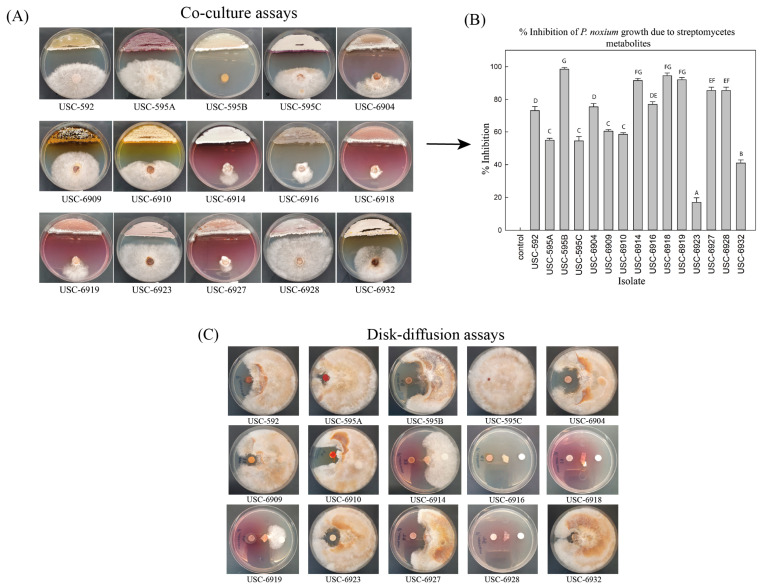
(**A**) Co-culture assays of the selected 15 termite gut-associated *Streptomyces* isolates and the *P. noxium* pathogen. (**B**) Percent inhibition of *P. noxium* mycelia when grown in co-culture with *Streptomyces* isolates. (**C**) Disk-diffusion assays of the *Streptomyces* isolate extracts against *P. noxium* (the left disk contains the active extracts, and the right disk is the control and contains EtOAc).

All 37 actinomycete isolates underwent a preliminary evaluation for antifungal activity against *P. noxium* using the co-culture assay technique (Table S2). From this, 15 actinomycete isolates demonstrated strong antifungal activity against the fungal pathogen (Figure 1A). The mean inhibition percentages are demonstrated in Figure 1B. The results showed significant differences between the control and co-culture plates (F (14, 60) = 152.880) *p* ≤0.001) with isolate USC-595B presenting with the strongest antifungal activity with a mean value of 98.5% mycelial inhibition. This was followed by USC-6918 with 94.5% and USC-6919 with 92.0% inhibition, respectively. These active isolates might therefore be a valuable reservoir of bioactive compounds with antifungal properties. As per the phylogenetic analysis, the 15 isolates demonstrating the potent antifungal activity showed clustering within the genus *Streptomyces*.

### 2.3. Antifungal Activity from Streptomycetes Fermentation Extracts against P. noxium

**Figure 2:**
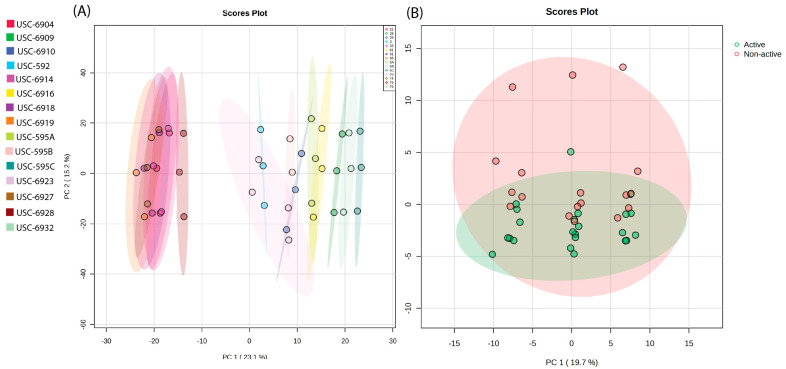
(**A**) Two-dimensional PCA score plot demonstrating the differential production of metabolites between the fifteen *Streptomyces* isolates across the positive and negative ionisation modes (PC1 × PC2) and (**B**) a score plot comparing the active and non-active fermentation extracts.

The 15 selected antifungal *Streptomyces* isolates underwent fermentation and extraction of their natural products. The resulting fermentation extracts were further tested for persistent antifungal activity using the Kirby-Bauer disk-diffusion method (Figure 1C). USC-595B demonstrated persistent strong antifungal activity, and USC-6916 and USC-6928 also displayed a substantial increase in antifungal activity compared with the initial co-culture assays (Figure 2A). The antifungal activity of some of the *Streptomyces* isolates, including USC-6904, USC-6927, USC-6910, and USC-6909, was reduced. This decrease might be due to the absence of antifungal volatile compounds or compound degradation during solvent extraction and evaporation. Based on these disk-diffusion activity assays, isolates presenting with the strongest activities and, therefore, of interest include USC-595B, USC-6904, UCS-6914, USC-6916, USC-6918, USC-6919, USC-6927, and USC-6928. These isolates and their closest related species can be linked to previous studies demonstrating antifungal activities. For instance, USC-595B was found to be most similar to *Streptomyces platensis*, which was identified as a strong antifungal strain [42,43,44]. Resormycin is an herbicidal and antifungal antibiotic isolated from *S. platensis*, which demonstrated activity against numerous fungal phytopathogens [45]. Wan et al. (2008) also found *S. platensis* to be an effective biocontrol agent for the suppression of many plant-pathogenic fungi, including *Botrytis cinerea*, *Rhizoctonia solani*, and *Sclerotinia sclerotiorum*. Additionally, biosynthetic gene clusters of Phoslactomycins (PLMs), which are polyketide natural products and important antifungals, were identified from *S. platensis* [46]. USC-6904 and USC-6914 were most similar to *S. catenulae*, which demonstrated antifungal activity against the fungal pathogen *P. citriasiana* [47]. USC-6916 was most similar to *S. gelaticus*, which was assessed for antifungal activity against basal stem rot caused by *Ganoderma boninense* using co-culture assays and found to produce up to 100% inhibition. The subsequent crude extracts generated from this strain demonstrated activity of around 87.5% inhibition from a 10% concentration [48]. USC-6918 was most similar to *S. luozhongensis*, which was found to have antifungal activity against *Saccharomyces cerevisiae*, *Aspergillus flavus*, *A. niger*, *Penicillium citrinum*, and *Candida albicans* [49]. USC-6919 was most similar to the thermophilic *S. thermocarboxydus*, which was shown to be a promising biocontrol agent against strawberry anthracnose caused by *Glomerella cingulate* [50]. USC-6927 was most similar to *S. alni* and was found to have antifungal activity against the root rot of grapevine [50]. USC-6928 was most similar to *S. diastaticus*, which was found to produce the macrolide antibiotics oligomycin A and C, which exhibited strong activity against *A. niger*, *Alternaria alternata, B. cinerea*, and *Phytophthora capsica* [51]. This further exemplifies the biopotential of these isolates to produce antifungal secondary metabolites for the purposes of biocontrol.

### 2.4. Metabolite Diversity Analysis of Streptomycetes Fermentation Extracts Using Untargeted MS-Based Metabolomic Approach

Principal component analysis (PCA) of the MS data was generated by comparing the chemical diversity between the 15 streptomycete fermentation extracts (Figure 2A). The PCA demonstrated a clear clustering of samples, indicating spectral similarities between isolates USC-6904, USC-6914, USC-6918, USC-6919, USC-6927, and USC-6928. The remaining isolates had a clear distinction between this cluster but still displayed differences between each other. It can also be noted that USC-6923 displayed the largest metabolic diversity. Additionally, USC-595A and USC-6916 also displayed a degree of similarity in their metabolic profiles. The generated PCA in Figure 2B displays a comparison between the 8 identified active extracts and the remaining 7 non-active extracts. The non-active extracts show a larger chemical diversity; however, there is a great deal of overlap with the active extracts.

### 2.5. Molecular Networking and Identification of Bioactive Compounds within the Streptomycetes Fermentation Extracts

**Figure 3:**
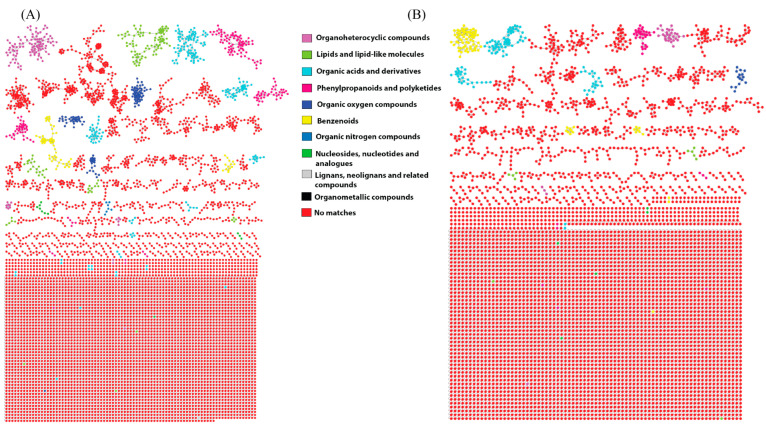
Termite gut-associated streptomycetes molecular network coloured by 11 chemical class terms as indicated by the legend. Chemical class annotation was performed using MolNetEnhancer within the GNPS framework (**A**) class annotated MN in a positive ionisation mode and (**B**) class annotated MN in a negative ionisation mode.

A molecular network (MN) was generated for the positive and negative electrospray ionisation mode datasets to detect as many compounds as possible. The MN of the positive dataset consisted of 6994 nodes, of which 114 were annotated by the GNPS spectral library database (Figure 3A). The negative dataset comprised 5629 nodes, and 38 nodes were annotated (Figure 3B). Each node represented a metabolite based on its molecular ion signal, and the edges connecting them represented the similarity of MS/MS fragmentation patterns among the nodes. To further enhance chemical structural information within the MN, the in silico structure annotation from the GNPS library search was incorporated into the network using the MolNetEnhancer workflow, which allowed for chemical class annotations using ClassyFire chemical ontology. It is apparent that the termite gut-associated streptomycetes produce structurally diverse compounds both between ionisation modes and between the individual samples (Figure 3). The identified chemical classes include organoheterocyclic compounds, lipids and lipid-like molecules, organic acids and derivatives, phenylpropanoids and polyketides, organic oxygen compounds, benzenoids, organic nitrogen compounds, nucleotides/sides, and lignans and neolignans (Figures S2 and S3). The most abundant chemical classes observed in node clusters include organic oxygen compounds, phenylpropanoids and polyketides, organic acids and derivatives, and lipids and lipid-like molecules. However, a majority of the metabolites visualised in the MN did not have a match to the chemical class annotations due to a lack of annotations from the GNPS library, indicating potential novel metabolites.

Streptomycetes are known to synthesise various antimicrobial compounds belonging to chemical classes such as aminoglycosides, peptides, ansamycins, β-lactams, tetracyclines, macrolides, lincosamides, epoxides, polyketides terpenoids, flavones, and aminocoumarins [52,53]. Many of these described chemical classes were also identified within the molecular network of the fermentation extracts of termite gut-associated *Streptomyces* isolates. Polyketides constitute a substantial and diverse group of secondary metabolites that display significant structural variability and versatile biological functions [54]. Beyond their pharmaceutical significance, actinomycete polyketides have also gained much interest in crop protection. Examples of these actinomycete polyketide pesticides include avermectins, milbemycins, and spinosyns, all of which offer a form of protection for plants against various pathogens and have been successfully commercialised and used in the agricultural field [55]. As seen in Figure 3, polyketide metabolites are among the most dominant observed in the metabolome of the termite gut-associated streptomycetes. Accordingly, they were selected for further investigation.

#### 2.5.1. Isoflavonoids

**Figure 4:**
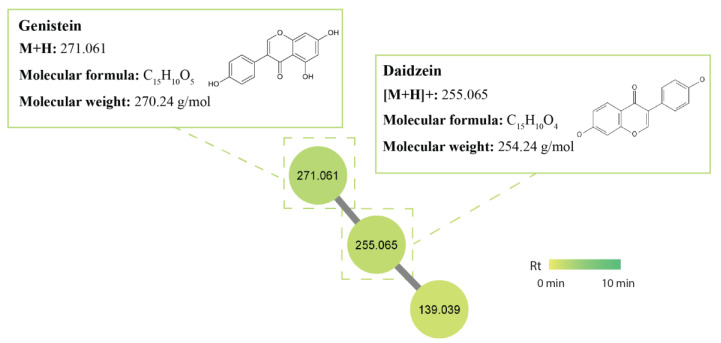
Isoflavonoid metabolite cluster identified in the termite gut-associated streptomycetes molecular network. Nodes represent an individual metabolite; each node displays its parent mass and is coloured based on its retention time.

Flavonoids and isoflavonoids are versatile natural products with extensive pharmacological activities. They have extremely low toxicity, making them compounds of interest and a “hotspot” for drug discovery and development [56]. However, research on flavonoids has favoured plant sources, resulting in relatively less focus on microorganisms as potential sources for novel flavonoids [56,57,58]. Within this class, a cluster of isoflavones, including daidzein (m/z 255.065 [M+H]^+^) and genistein (m/z 271.061 [M+H]^+^), were tentatively identified within the GNPS library (Figure 4). One study found that 10% of their 200 tested soil *Streptomyces* isolates produced daidzein and genistein and that genistein increased the production of polyketide antibiotics by 11–42% within its fermentation extracts [59]. The potential discovery of new isoflavonoids may be found in termite-associated streptomycetes as a study found three new isoflavonoid glycosides, which were isolated from termite-associated *Streptomyces* spp. recovered from the cuticle of South African termites, and as such were named termisoflavones A-C. These isolates were also found to produce daidzein and genistein [60]. Derivatives of daidzein and genistein, 7-O-β-D-glucosyl-genistein and 7-O-β-D-glucosyl-daidzein, have also demonstrated considerable antifungal activity against *P. citricarpa*, meaning there may be potential for antifungal activity against *P. noxium* [61]. These two compounds were produced in isolates USC-595C, USC-595A, USC-6928, and USC-6916.

#### 2.5.2. Macrolides

**Figure 5:**
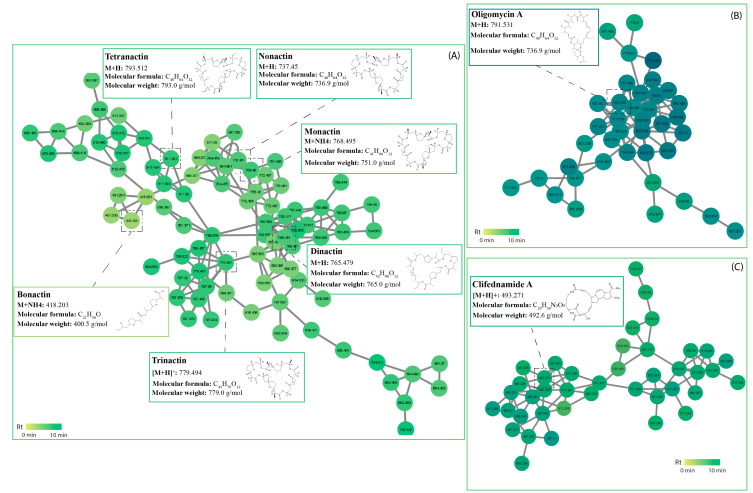
Macrotetrolide cluster identified in the termite gut-associated streptomycetes molecular network. Nodes represent an individual metabolite; each node displays its parent mass and is coloured based on its retention time. (**A**) macrotetrolides (**B**) macrocyclic lactones (**C**) polycyclic tetramate macrolactums.

Macrolides were originally isolated from *Streptomyces* species and are antibiotics consisting of a macrocyclic lactone ring, often 14-, 15- or 16-membered ring compounds with which various amino sugars are attached [62]. The most common macrolides are oleandomycin isolated from *Streptomyces antibioticus*, spiramycin isolated from *S. ambofaciens,* and erythromycin isolated from *S. erythreus*. Recent findings of new macrolide antibiotics from *Streptomyces* species have demonstrated potent antifungal activity. For instance, nine new polyene macrolides were isolates from deep-sea-derived *Streptomyces antibioticus* that demonstrated antifungal activity against *Candida albicans* [63]. Additional four new macrolides named venturicidins G-J were isolated from *Streptomyces* sp. *SN5452*, exhibiting strong inhibition towards *Pyricularia oryzae*, which causes the destructive rice blast disease [53]. A cluster of macrotetrolide antibiotics were also tentatively identified within this class, including nonactin (m/z 737.450 [M+H]^+^), monactin (m/z 768.495 [M+NH4]^+^), dinactin (m/z 765.481 [M+H]^+^), trinactin (m/z 779.494 [M+H]^+^), bonactin (m/z 418.203 [M+NH4]^+^), and tetranactin (m/z 793.512 [M+H]^+^), all found in isolates USC-595A and USC-595C (Figure 5). These macrolides are also known to be produced by a variety of *Streptomyces* spp., particularly by *S. globisporus* [64]. They exhibit antibacterial and antifungal activity as well as acaricidal, insecticidal, coccidiostatic, and anthelminthic activities [65]. One study investigated the bioactive compounds of *S. araugoniae* that proved effective against many phytopathogenic fungi and found a complex of macrotetrolides that included these compounds, all of which presented with antifungal activity against *Botrytis cinerea* [66]. Another study found that dinactin had broad inhibitory activity towards multiple phytopathogenic fungi, including *Colletotrichum gloeosporioides* [67]. A macrolide antibiotic within a separate cluster was identified as oligomycin A (m/z 791.531 [M+H]) and is reported to be produced by some strains of *Streptomyces*. They have a broad spectrum of activities against organisms such as fungi, bacteria, and nematodes. Oligomycin was first described as a new antifungal antibiotic in *Streptomyces diasatochromogenes* with nine isomers identified soon after [68]. A study conducted by Chakraborty (2020) investigated oligomycins produced by streptomycetes and evaluated the in vitro suppressive effects on hyphal growth, condidogenesis, conidial germination, and appressorial formation of the wheat blast fungus *Magnaporthe oryzae* and found strong inhibitory activity across all areas [69]. This oligomycin cluster was found in isolates USC-592, USC-595A, USC-6923, and USC-6928 (Figure 5). A separate phenylpropanoid and polyketide cluster identified as polycyclic tetramate macrolactum (PTM) clifednamide A (m/z 493.271 [M+H]^+^) is produced by streptomycetes and other bacteria. PTMs are a fast-growing antibiotic natural product family with diverse therapeutic properties, such as antibacterial, antifungal, antiprotozoal, and anticancer [70]. It has also been suggested that the fungicidal action of macrolide antibiotics is their affinity for binding to ergosterol within fungal membranes [71].

#### 2.5.3. Coumarins

**Figure 6:**
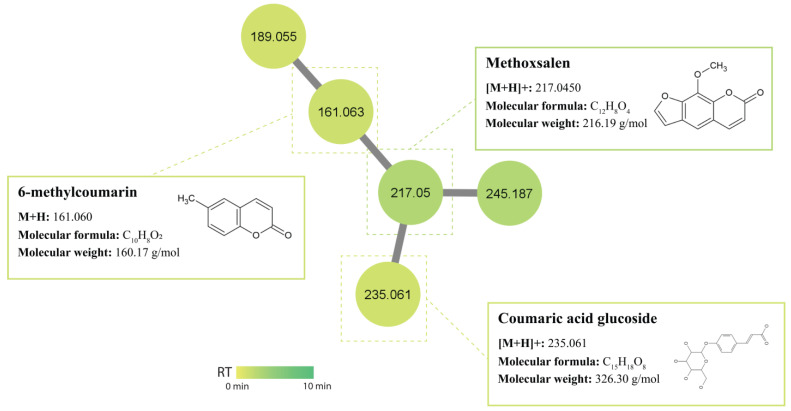
Coumarin cluster identified in the termite gut-associated streptomycetes molecular network. Nodes represent an individual metabolite; each node displays its parent mass and is coloured based on its retention time.

Coumarin and its derivatives represent one of the most active classes of compounds possessing biological activities [72], such as antibacterial, antifungal, antiviral, and anticancer [73]. Since the discovery of novobiocin (an aminocoumarin antibiotic) produced by *Streptomyces niveus*, coumarins have attracted more attention as potential antimicrobial agents. The GNPS library displayed a match to 6-methylcoumarin (m/z 161.060 [M+H]) produced by isolates USC-6932 and USC-6904. In a study conducted by Chen (2022), the antifungal activity of coumarins against phytopathogenic fungi was evaluated and found that 6-methylcoumarin presented with the strongest antifungal activity against Valsa mali [74]. Another coumarin was manually annotated as methoxsalen (m/z 217.045 [M+H]^+^) produced by isolates USC-592 and USC-6932 (Figure 6). Methoxsalen (xanthotoxin) is a photosensitising furocoumarin compound and has been used as the first treatment of mycosis fungoides [75].

#### 2.5.4. Cyclic Peptides (Nocardamine)/Siderophores

**Figure 7:**
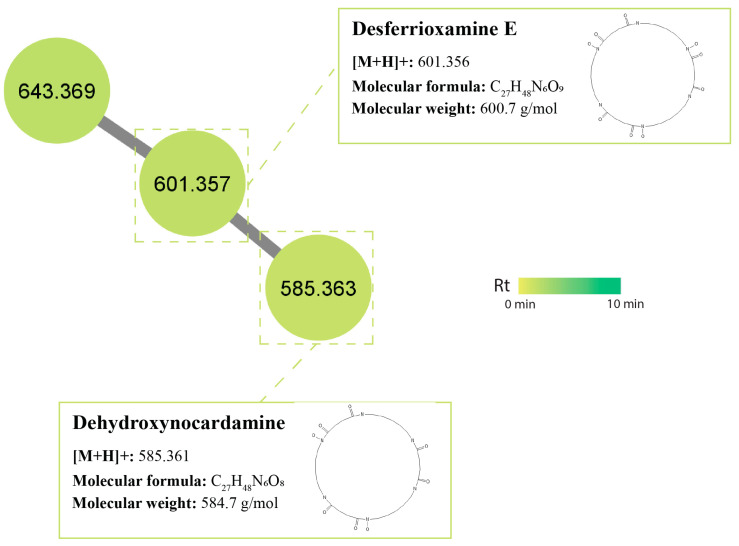
Peptide cluster identified in the termite gut-associated streptomycetes molecular network. Nodes represent an individual metabolite; each node displays its parent mass and is coloured based on its retention time.

Desferrioxamines are the principle siderophores produced by *Streptomyces* species [76]. Studies have shown that streptomycetes are able to use their siderophore production as a means to ‘starve’ fungi of iron [77] as an antagonistic mechanism. A cluster of siderophore peptides was also identified within the MN, including desferrioxamine E (synonym: nocardamine) (m/z 601.356 [M+H]^+^) which is a siderophore widely produced by *Streptomyces* species [76] and was found in isolates USC-595A and USC-592. Derivatives of this compound, also found in streptomycetes and identified as dehydroxynocardamine (m/z 585.631 [M+H]^+^), were found in isolate USC-592 (Figure 7).

### 2.6. Pathway Enrichment Analysis within the Streptomycetes Fermentation Extracts

Pathway enrichment analysis (PEA) and overrepresentation analysis (ORA) play vital roles in high-dimensional molecular data and are becoming popular methods for analysing metabolic data as these include specific fingerprints of cellular biochemistry [78,79]. These analyses were conducted to investigate the pathways that were amplified across all 15 streptomycete extracts and how they may support the metabolite identification within the molecular networks of this study. The PEA revealed multiple pathways of interest due to their role in antibiotic synthesis. These include streptomycin biosynthesis, polyketide sugar unit biosynthesis, monobactam synthesis, siderophore group nonribosomal peptides synthesis, terpenoid backbone synthesis, biosynthesis of secondary metabolites, streptomycin synthesis, and novobiocin synthesis. Similarly, the ORA demonstrated enriched metabolite sets of interest, including (i) purine metabolism; (ii) phenylalanine, tyrosine, and tryptophan synthesis; and (iii) neomycin, kanamycin, and gentamicin biosynthesis.

Purine metabolism plays a crucial role in supplying energy and essential precursors for antibiotic biosynthesis in *Streptomyces* spp. [80]. Furthermore, it was reported that phenylalanine deaminates to trans-cinnamic acid, which ultimately can be converted into secondary metabolites, such as lignins, flavonoids, and coumarins, all of which were identified within the molecular network in this study [58,81]. One study investigated an endophytic *Streptomyces* isolate and discovered antibiotics kanamycin and gentamicin within their metabolomic analysis [82,83]. The ORA supports the detected metabolites in the molecular network and validates key compounds such as flavonoids and courmarins.

**Figure 8:**
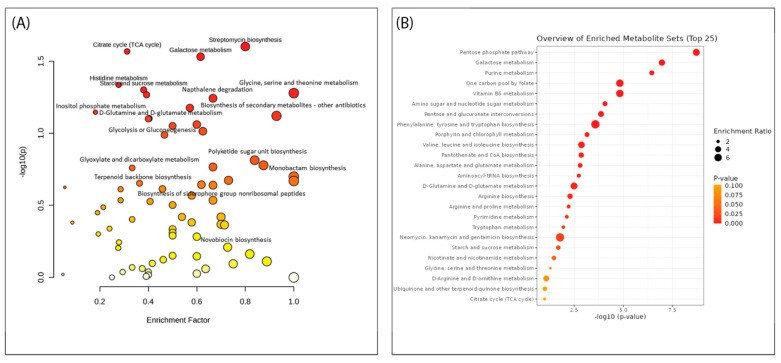
(**A**) Pathway enrichment and (**B**) overrepresentation analyses generated in Metaboanalyst 5.0 of all 15 of the *Streptomyces* isolates from the MS data. The size of the circle is indicative of the pathway impact score, while the colours are based on the *p*-value (the darker the colour, the higher the significance of the metabolites within the relevant pathway).

Important pathways responsible for secondary metabolite production include polyketide synthases (PKS) type I, II, and III, as well as nonribosomal peptide synthase (NRPS), both of which were identified within the PEA of this study. PKS genes encode polyketide metabolites such as antifungal antibiotic amphotericin B [84]. Researchers have reported the biosynthesis behind macrocyclic polyketides (macrolide antibiotics) by investigating the encoded PKS pathway. They revealed that these products are synthesised from acetate, propionate, and other short-chain fatty acids within this pathway and, thus, form a hydroxylated macrocyclic lactone ring with three to seven conjugated double bonds and one or more sugars [85,86]. Many of the enriched pathways support the finding presented in the MN and the possibility of the biosynthetic potential of these termite gut-associated *Streptomyces* in producing novel antifungal antibiotics(Figure 8).

## 3. Materials and Methods

### 3.1. Cultures and Culture Media

The 37 actinomycete isolates screened in this study were previously isolated from the guts of termites, *Coptotermes lacteus* (Froggatt) species located in the Sunshine Coast Region of Queensland, Australia [36,37], and cryogenically preserved in the University of the Sunshine Coast Microbial Library. The actinomycetes were grown onto oatmeal agar [87], and the *P. noxium* strain was previously isolated from infected heritage fig trees within Brisbane, Queensland, and grown on potato dextrose agar (PDA) (OXOID, Australia).

### 3.2. 16S rDNA Oligonucleotide Sequencing of Actinomycete Isolates

Extraction and amplification of the 16S rDNA were carried out using a commercial QIAGEN HotStarTaq DNA PCR kit as per the manufacturer’s instructions and using the eubacterial universal primers 27F 5′ -AGAGTT TGA TCC TGG CTC AG-3′ and 1492R 5′-GGT TAC CTT GTT ACG ACT T-3′. Conventional PCR reactions were performed achieving a final volume of 50 µL; 2× PCR master mix 25 µL, 10× primer mix 5 µL, Q-solution 10 µL, and RNase-free water 10 µL. Amplification was performed using the Bio-Rad T100 Thermocycler, (McHugh Electronic, Australia) with thermocycling conditions beginning with the initial temperature of 95 °C for 15 min, followed by 35 cycles of 94 °C for 1 min, 68 °C for 1 min, and final temperature of 72 °C for 10 min. PCR products were electrophoresed on a 1.5% agarose gel followed by visual confirmation under an IV transilluminator. The PCR products were sent to Macrogen (Seoul, Republic of Korea) (http://dna.macrogen.com/eng/, accessed on 22 November 2021) for sequencing. The sequences were analysed using Geneious Prime 23023.1.2 and putative identifications were made by conducting a BLAST (Basic Local Alignment Search Tool) search using the NCBI (National Centre for Biotechnology Information) database. This database is also where the sequences generated from this study have been deposited. Reference and generated accession numbers can be found in the Supplementary Materials.

### 3.3. Evaluation of Antifungal Activity of the Actinomycete Isolates

The 37 actinomycete isolates were screened for antifungal activity against the fungal pathogen *P. noxium* using co-culture assays. The isolates were streaked at one end of the agar plate and incubated for 7 days to grow at 28 °C before a fungal plug was placed at the opposing end of the plate. A control plate was also established without the *Streptomyces* isolates. Once the control had reached the edge of the plate, comparisons were made using the following formula:

$$Inhibition\;Zone\;\left(\%\right)=\sum \left(\frac{Colony\;diameter\;of\;pathogen-\left(colony\;diameter\;of\;pathogen+antagonist\right)}{Colony\;diameter\;of\;pathogen}\right)\times 100$$

### 3.4. Fermentation and Extraction of Metabolites Produced by Streptomyces Isolates

Fermentation of the *Streptomyces* isolates was conducted using solid-state fermentation. The isolates were inoculated onto oatmeal agar plates and incubated for 7 days at 28 °C. The plates were then inoculated with a fungal plug from the *P. noxium* and grown for three more days using the same conditions. These plates were then twice extracted using ethyl acetate (ChemSupply) (EtOAc) by cutting up the agar and flooding them with the solvent for 24 h. The solvent was filtered and evaporated under a vacuum to obtain the crude extract for further testing.

### 3.5. Bioassays of the Streptomycetes Fermentation Extracts Using Disk-Diffusion Method

The antifungal activity of the crude extracts was then assessed using the Kirby-Bauer disk-diffusion method [88]. Sterile paper disks were seeded with the crude extract (approx. 100 µL) and placed onto PDA plates (2 cm away from the edge) (left). A blank control disk was also added, containing only DMSO on the opposing side (right). A fungal plug from the fungal pathogen was placed onto the centre of the plate and was incubated at 25 °C for four days. Signs of inhibition of the fungal mycelium were then observed.

### 3.6. QTOF Chemical Profiling of the Metabolites within the Streptomyces Fermentation Extracts

Liquid chromatography with accurate mass spectroscopy (LC-MS) was performed on a SCIEX ExionLC system coupled to a SCIEX X500 QTOF. The column was a Synergi Fusion RP, 50 mm × 4.6 mm, 2.5 µm. Mobile phase A was 99.9% MilliQ water with 0.1% formic acid, and mobile phase B was 99.9% LC-grade methanol with 0.1% formic acid. The mobile phase program was 1.0 mL/min commencing at 98:2 MPA: MPB for 0.25 min, grading to 100% MPB at 6.25 min, isocratic until 8.25 min, grading back to 98:2 MPA:MPB at 8.5 min, and isocratic until 10.0 min. The mass spectrometer was operated in alternating positive and negative electrospray ionisation modes, providing accurate M+1 and M-1 molecular ions to four decimal places.

### 3.7. MS Data Analysis and Annotation

The data were initially converted from “.wiff” standard file format to “.mzXML” using MSconvert within the ProteoWizard 3.0 software toolkit. The data were then processed using MZmine 2.53 and exported as a .CSV file where it was analysed using MetaboAnalyst 5.0 (https://new.metaboanalyst.ca, accessed on 22 November 2021), which allowed for the generation of principal component analysis (PCA), pathway enrichment analysis (PEA), and overrepresentation analysis (ORA).

A molecular network was generated using the online workflow (https://ccms-ucsd.github.io/GNPSDocumentation/, accessed on 22 November 2021) on the GNPS website (http://gnps.ucsd.edu, accessed on 22 November 2021) (Wang et al., 2016) [89]. The precursor ion mass tolerance was set to 2.0 Da and a MS/MS fragment ion tolerance of 0.5 Da. A network was then created where edges were filtered to have a cosine score above 0.65 and more than 4 matched peaks. Further, edges between two nodes were kept in the network if and only if each of the nodes appeared in each other’s respective top 10 most similar nodes. Finally, the maximum size of a molecular family was set to 100, and the lowest-scoring edges were removed from molecular families until the molecular family size was below this threshold. The resulting network was visualized in Cytoscape. Annotations of the metabolites were first made by matching the spectra against GNPS’ spectral libraries. All matches between network spectra and library spectra were required to have a score above 0.7 and at least 4 matched peaks. Generated annotations were validated against the Metabolomics Standards Initiative (MSI) [90] and were in compliance with level 2 annotations corresponding to “putatively annotated compounds” [91].

## 4. Conclusions

In this study, the phylogeny and antifungal activity of termite gut-associated actinomycetes were investigated. The 16S rRNA gene analysis revealed a dominance of the members of the genus *Streptomyces* in the termite gut. Additionally, 15 of the *Streptomyces* isolates presented with strong antifungal activity against the phytopathogenic fungus *P. noxium*. Fermentation extracts of the selected isolates were analysed through the employment of MS-based metabolomics for the evaluation of their metabolome chemical composition. Known antifungal metabolites of the polyketide chemical class were identified, including isoflavonoids, macrolides, coumarins, and cyclic peptides. Pathway enrichment analyses supported the biosynthetic potential for antibiotic production by the *Streptomyces* isolates and the findings of the molecular networking. The results of this study provide insights into the chemical composition and biopotential of termite gut-associated *Streptomyces* isolates as unexplored sources for novel antifungal compounds for biocontrol applications.

## Data Availability

Data are contained within the article or Supplementary Material.

## Supplementary Materials

The following supporting information can be downloaded at: https://www.mdpi.com/article/10.3390/antibiotics12091373/s1, Table S1: Termite gut-associated actinomycetes phylogeny; Table S2: Assigned GenBank accession numbers of the actinomycete isolates; Figure S1: Phylogenetic tree of the actinomycetes; Figure S2: Metabolite chemical class distribution; Figure S3: Distribution of polyketide metabolites across the fermentation extracts of streptomycetes.
