# Supplementary material for: Untargeted MS-Based Metabolomic Analysis of Termite Gut-Associated Streptomycetes with Antifungal Activity against Pyrrhoderma noxium
Source: Antibiotics (Basel). 2023 Aug 28;12(9):1373. doi: 10.3390/antibiotics12091373 (PMC10525753; doi:10.3390/antibiotics12091373)
Supplement: Supplementary file 1 [file antibiotics-12-01373-s001.zip › antibiotics-2570570-supplementary.pdf]

Untargeted MS-based Metabolomic Analysis of Termite Gut-Associated Streptomycetes with Antifungal Activity against *Pyrrhoderma noxium*

Cherrihan Adra [1], Trong D. Tran [2], Keith Foster [3], Russell Tomlin [3] and D. İpek Kurtböke [1,*]

[1] School of Science, Technology and Engineering, University of the Sunshine Coast, Maroochydore BC, QLD 4558, Australia; cherrihan.adra@research.usc.edu.au (C.A.); ttran1@usc.edu.au (T.D.T.)
[2] Centre for Bioinnovation, University of the Sunshine Coast, Sippy Downs, QLD 4556, Australia
[3] Brisbane City Council, Program, Planning and Integration, Brisbane Square, Level 10, 266 George Street, Brisbane, QLD 4000, Australia; kfosteradsl@bigpond.com (K.F.); russell.tomlin@brisbane.qld.gov.au (R.T.)
* Correspondence: ikurtbok@usc.edu.au

Supplementary material

GNPS molecular networking job link: ID=61d105da9f264f26be65600a18d777b7

Tables:

Table S1. Termite gut-associated actinomycete isolates phylogeny and morphology

Strain code	Closest related species	%Identity	Reference Accession number	Morphology
USC-590	*Micromonospora chalcea* strain IMB16-203	100%	MG190678.1	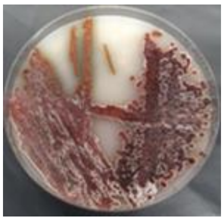
USC-591	*Micromonospora chalcea* strain IMB16-203	99.92%	MG190678.1	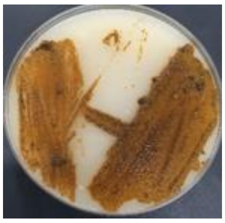
USC-592	*Streptomyces albiaxialis* strain VLK-10	100%	KP170480.1	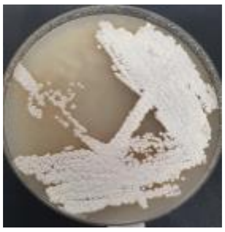

USC-593	*Micromonospora chalcea* strain IMB16-203	100%	MG190678.1	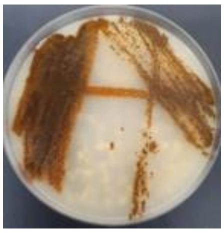
USC-595A	*Streptomyces puniceus* strain IIPR:KR01:01	100%	MK825538.2	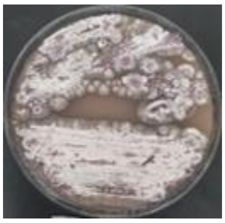
USC-595B	*Streptomyces platensis* strain HBUM174787	100%	FJ486385.1	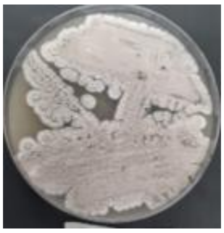
USC-595C	*Streptomyces diastaticus* strain FJAT-31547	100%	MH084838.1	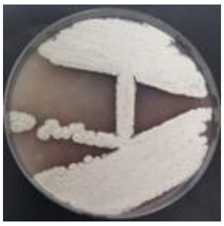
USC-596	*Streptomyces laurentii* strain SNP3	99.92%	MZ389926.1	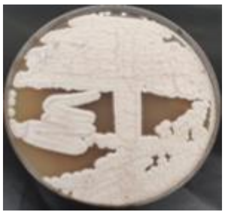
USC-597	*Streptomyces cirratus* strain BS32	100%	KR063212.1	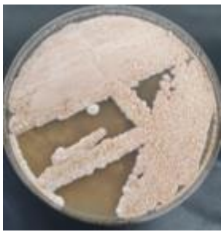

USC-598	*Streptomyces sp.* strain ZMR2-66	100%	MZ833414.1	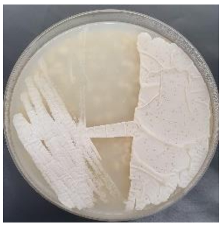
USC-599	*Micromonospora chalcea* strain IMB16-203	99.76%	MG190678.1	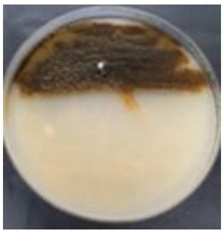
USC-6902	*Streptomyces paucisporeus* strain WZ092-100	98.25%	JF723499.1	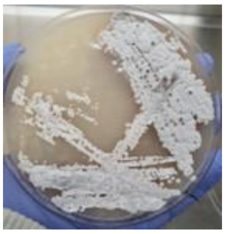
USC-6902A	*Streptomyces* sp. B424	99.20%	KJ781978.1	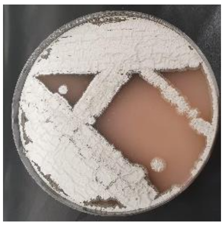
USC-6903	*Streptomyces puniceus* strain NBRC 12811	100%	MK424311.1	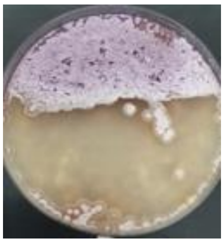
USC-6904	*Streptomyces catenulae* strain NBRC 12848	99.76%	NR_112303.1	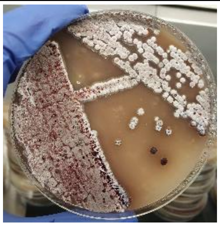

USC-6905	*Streptomyces fradiae* strain ES1	100%	MG266050.1	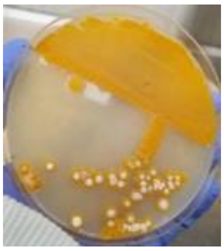
USC-6906	*Saccharopolyspora erythraea* strain NRRL 23338	99.92%	CP069353.1	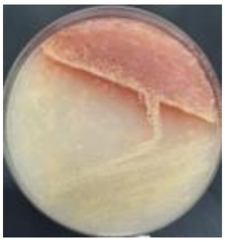
USC-6907	*Streptomyces* sp. strain HBUM206419	100%	MT540570.1	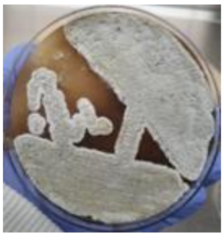
USC-6909	*Streptomyces* sp. strain ZMR2-66	100%	MZ833414.1	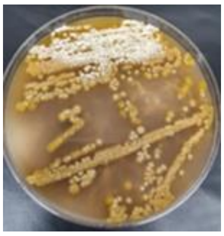
USC-6910	*Streptomyces* sp. strain Ecaf-6	100%	MK764948.1	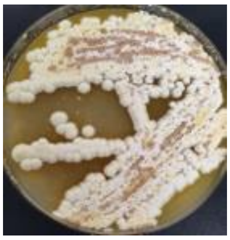
USC-6911	*Streptomyces diastaticus* subsp. ardesiacus strain 205519-2	100%	MH998230.1	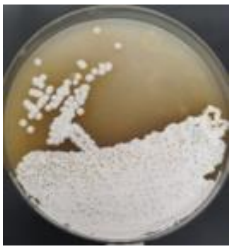

USC-6914	*Streptomyces catenulae* strain NBRC 12848	99.92%	NR_112303.1	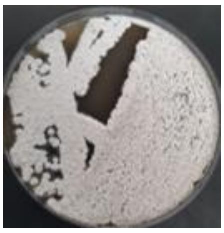
USC-6916	*Streptomyces* sp. strain TM-A140	100%	MH698641.1	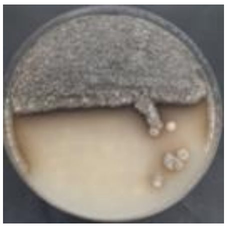
USC-6917	*Micromonospora* sp. strain HNM0611	100%	MN446732.1	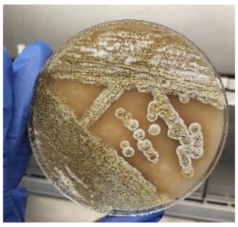
USC-6918	*Streptomyces luozhongensis* strain TRM49605	100%	NR_164875.1	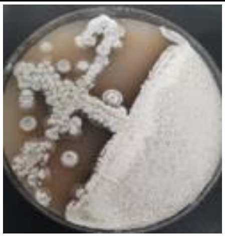
USC-6919	*Streptomyces thermocarboxydus* strain EGI124	100%	MN704433.1	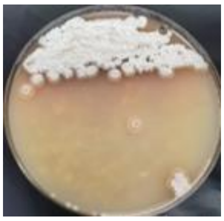
USC-6921A	*Streptomyces laurentii* strain T2-24	100%	OL584412.1	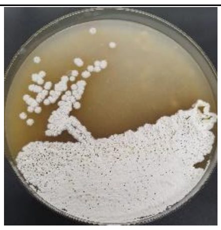

USC-6922	*Streptomyces laurentii* strain T2-24	100%	OL584412.1	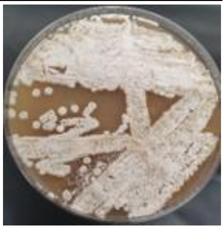
USC-6923	*Streptomyces* sp. strain CX3	100%	MN636760.1	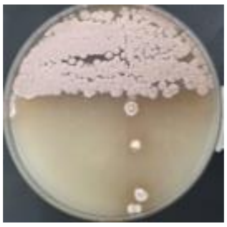
USC-6924	*Streptomyces* sp. Ank245	99.68%	HQ662223.1	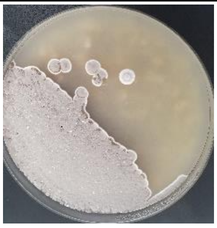
USC-6925	*Streptomyces drozdowiczii* DGA8-3	100%	LC536754.1	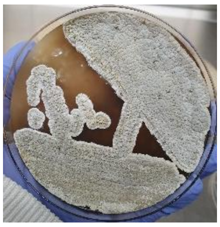
USC-6927	*Streptomyces alni* strain D65	100%	NR_043866.1	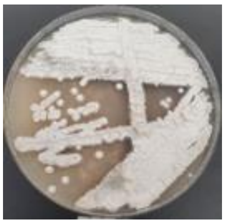
USC-6928	*Streptomyces diastaticus* strain FJAT-31547	100%	MH0848381.1	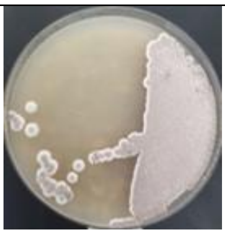

USC-6930	*Streptomyces costaricanus* strain K21C32	99.92%	MT124459.1	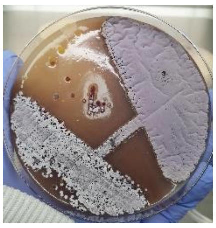
USC-6932	*Streptomyces* sp. GX9	100%	JF830633.1	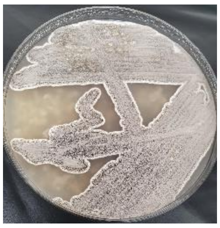
USC-6935	*Streptomyces fumigatiscleroticus* strain XY191	97.62%	MH432668.1	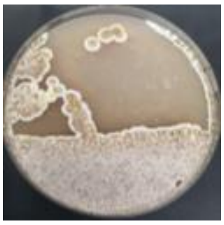
USC-6953	*Streptomyces cirratus* strain HW276-2	100%	ON738616.1	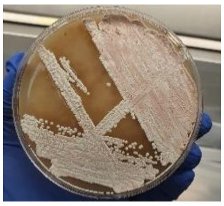

Table S2. Assigned GenBank accession numbers of the actinomycete isolates

Strain Code	GenBank accession numbers
USC-590	OQ740099
USC-591	OQ740100
USC-592	OQ740101
USC-593	OQ740102
USC-595A	OQ740103
USC-595B	OQ740104
USC-595C	OQ740105
USC-596	OQ740106
USC-597	OQ740107
USC-598	OQ740108
USC-599	OQ740109
USC-6902	OQ740110
USC-6902A	OQ740111
USC-6903	OQ740112
USC-6904	OQ740113
USC-6905	OQ740114
USC-6906	OQ740115
USC-6907	OQ740116
USC-6909	OQ740117
USC-6910	OQ740118
USC-6911	OQ740119
USC-6914	OQ740120
USC-6916	OQ740121
USC-6917	OQ740122
USC-6918	OQ740123
USC-6919	OQ740124
USC-6921A	OQ740125
USC-6922	OQ740126
USC-6923	OQ740127
USC-6924	OQ740128
USC-6925	OQ740129
USC-6927	OQ740130
USC-6928	OQ740131
USC-6930	OQ740132
USC-6932	OQ740133
USC-6935	OQ740134
USC-6953	OQ740135

Figures:

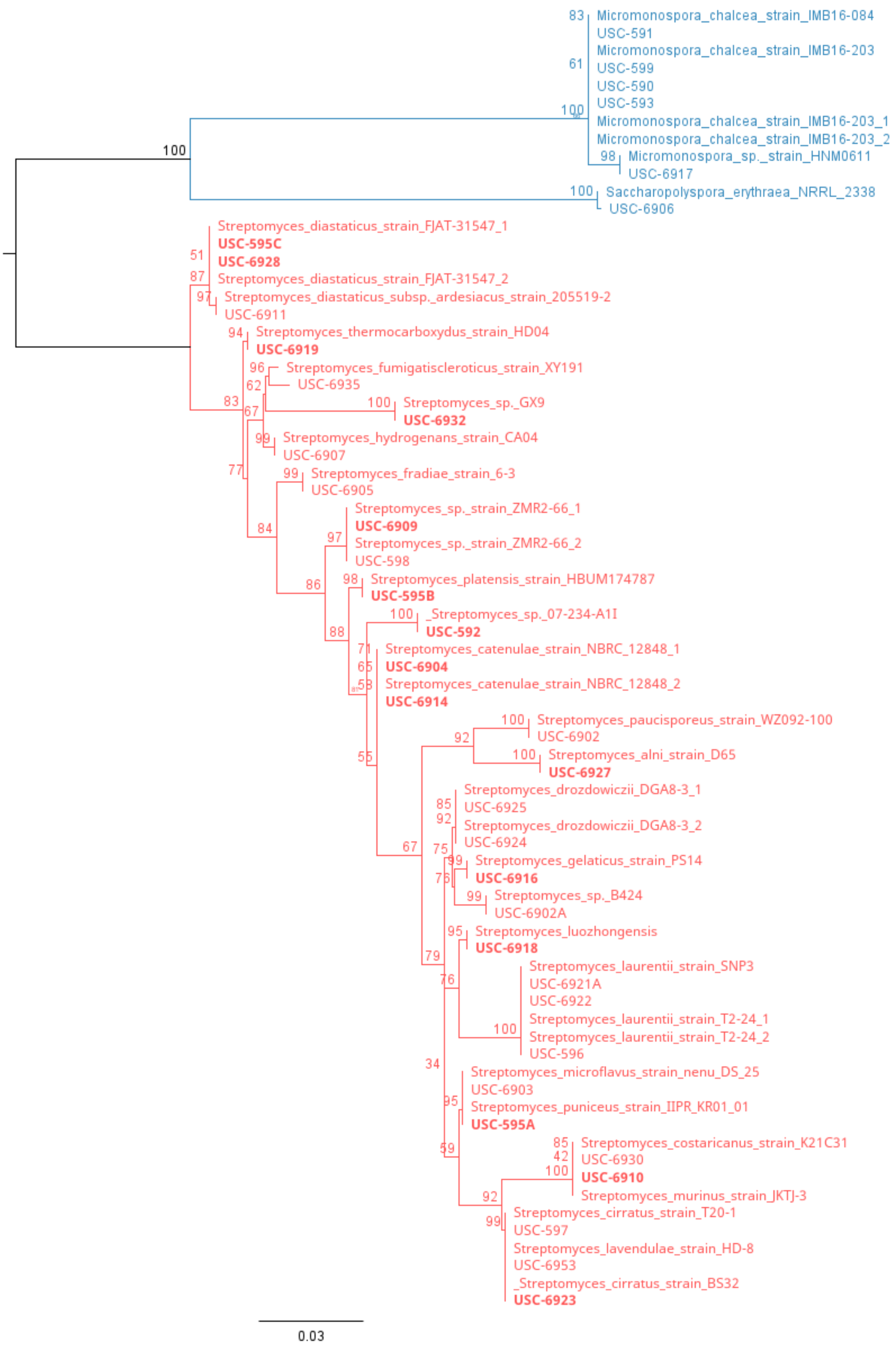

Figure S1. Phylogenetic relationship between actinomycetes previously isolated from the gut of termites and 37 global reference strains. The midpoint-rooted maximum likelihood phylogenetic tree was constructed using IQTREE2. The actinomycete sequences were named as per their corresponding GenBank accession number (Supplementary Materials) and coloured based on their genus. The second clade in red represents the genus *Streptomyces*, and the sequences investigated in this study are shown in bold. Bootstrap values are presented as a percentage, and the scale bar is indicative of the number of nucleotide substitutions per site.

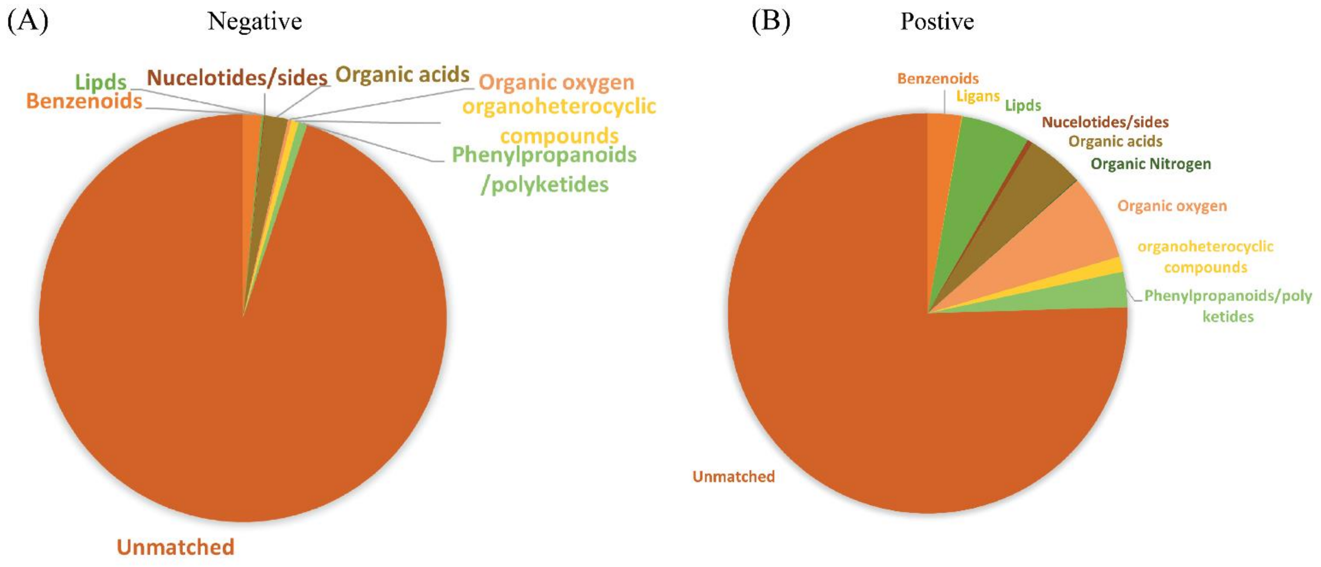

Figure S2. Metabolite chemical class distribution within the **(A)** positive molecular network and **(B)** negative molecular network.

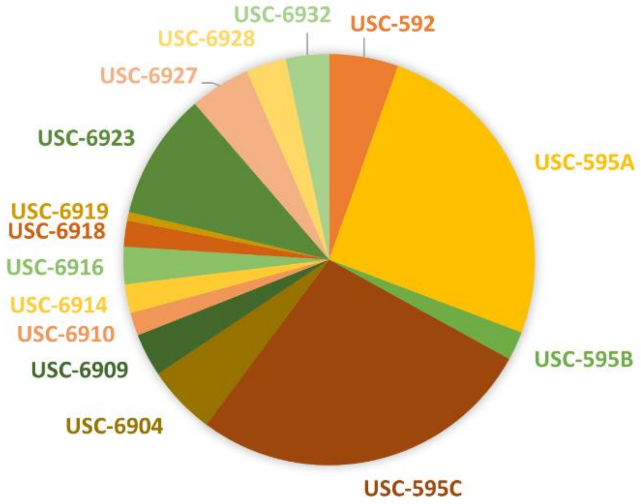

Figure S3. Distribution of polyketide metabolites across the fermentation extracts of streptomycetes.
